# Taking the diet of cows into consideration in designing payments to reduce enteric methane emissions on dairy farms

**DOI:** 10.3168/jds.2022-22766

**Published:** 2023-10

**Authors:** F. Le Gloux, S. Duvaleix, P. Dupraz

**Affiliations:** INRAE, Institut Agro, SMART, 35000 Rennes, France

**Keywords:** payment for environmental services, milk production, marginal cost, enteric emissions indicator

## Abstract

Enteric fermentation from dairy cows is a major source of methane. Significantly and rapidly reducing those emissions would be a powerful lever to mitigate climate change. For a given productivity level, introducing fodder with high sources of n-3 content, such as grass or linseed, in the feed ration of dairy cows both improves the milk nutritional profile and reduces enteric methane emissions per liter. Changing cows' diet may represent additional costs for dairy farmers and calls for the implementation of payments for environmental services to support the transition. This paper analyzes 2 design elements influencing the effectiveness of a payment conditioned toward the reduction of enteric methane emissions: (1) the choice of emission indicator capturing the effect of farmers' practices, and (2) the payment amount relative to the additional milk production costs incurred. Using representative farm-level economic data from the French farm accountancy data network, we compare enteric methane emissions per liter of milk calculated with an Intergovernmental Panel on Climate Change Tier 2 method, to baseline emissions from a Tier 3 method accounting for diet effects. We also quantify the additional milk production costs of integrating more grass in the fodder systems by estimating variable cost functions for different dairy systems in France. Our results show the relevance of using an emission indicator sensitive to diet effects, and that the significance and direction of the additional costs for producing milk with a diet containing more grass differ according to the production basin and the current share of grasslands in the fodder crop rotation. We emphasize the importance of developing payments for environmental services with well-defined environmental indicators accounting for the technical problems addressed, and the need to better characterize heterogeneous funding requirements for supporting a large-scale adoption of more environment-friendly practices by farmers.

## INTRODUCTION

The agricultural sector is a major source of greenhouse gases (**GHG**), accounting for 10% of EU-KPs (including the European Union, the United Kingdom, and Iceland under the Kyoto Protocol) in 2018 (Citepa, 2020a; EEA, 2020). It is estimated that 81% of agricultural methane emissions results from enteric fermentation, and 39% of that 81% is produced by dairy cows (EEA, 2020). Methane is the second contributor to radiative forcing. Currently, the global warming potential of methane is set at 28 times higher than the global warming potential of carbon dioxide over 100 years and 84 times higher over 20 years (Myhre et al., 2014). As methane is a short-lived climate pollutant continuously destroyed in the atmosphere, its effect on climate change depends mostly on short-term emission rates. In theory, decreasing the methane emissions rate below its natural destruction rate would have a cooling effect (Cain et al., 2019). Therefore, a significant reduction in methane emissions, in particular from agricultural activities, would rapidly mitigate climate change and is a powerful lever to meet the EU's 2050 climate targets (Dupraz, 2020).

Enteric fermentation is identified as the first source of GHG emissions from dairy farms in both developed and developing countries (Jayasundara et al., 2019; Wilkes et al., 2020). The quantity of methane produced during the digestive process of ruminants depends highly on the characteristics of the animal itself, such as the breed, body weight, and age (Gavrilova et al., 2019). However, enteric methane emissions are also directly related to farming practices, in particular the amount of feed intake and composition, and the proportion of carbohydrates that feed ration contains (Martin et al., 2010). In particular, for a given productivity level, enteric methane emissions decline as dairy cow feed is enriched with α-linolenic acid (ALA; polyunsaturated fatty acid of the n-3 family), for which the main natural sources are linseed and grass fodders (Dong et al., 1997; Martin et al., 2006, 2008, 2010, 2011; Chilliard et al., 2009; Grainger and Beauchemin, 2011). Moreover, as productivity per cow increases, methane emissions per kilogram of milk decrease (Martin et al., 2006). Animal productivity can also be improved through nutrition, as well as through herd management and genetics (Boadi et al., 2004). To accurately monitor the evolution of enteric methane emissions on dairy farms, one must consider both dimensions, productivity and feeding. Studies show that enteric methane emissions can differ significantly from one indicator to another and recommend considering both production intensity and feed usage for more accurate estimates (Hagemann et al., 2011; Sauvant et al., 2011).

Changing management practices, and in particular cows' diets, to decrease enteric methane emissions may be costly for many farmers. Economic incentives are currently being developed in the agricultural sector to support the reduction of GHG emissions. Although studies show that taxes and emission permits are the most efficient mitigation policy options, they are unpopular tools among producers and consumers (Key and Tallard, 2012; Douenne and Fabre, 2020; Funke et al., 2022). The agricultural sector remains exempt from carbon pricing schemes in most developed countries (Wirsenius et al., 2011; Henderson and Verma, 2021). In the EU, the agri-environmental policy has historically been designed as positive support instruments (agri-environmental schemes; Baylis et al., 2008). Alternatively, there is growing interest in financially supporting changes of management practices through payments for environmental services (**PES**), as more acceptable tools in the short term. Targeting enteric methane emissions is of primary interest for potential contributors, due to its high and rapid mitigation potential. In particular, supporting diet change levers, such as increasing grass use, is also highlighted by life cycle assessment approaches as having the potential to reduce total global warming potential and biodiversity damage of milk production (O'Brien et al., 2012; Guerci et al., 2013). Developing PES schemes for the reduction of enteric methane emissions raises the question of the choice of a practical emission indicator that is sensitive to both diet and productivity effects and that is easily applicable and measurable on farm. Although numerous other motivations may encourage farmers to join a PES program, such as improving milk quality, environmental quality, zootechnical performance, and the public image of agriculture, economic interests are likely to be crucial factors. An efficient payment level of a PES scheme targeting GHG emissions is equal to the socially optimal carbon price. In the EU, carbon prices differ widely among countries, illustrating the difficulty in pricing GHG emissions and therefore the social value of mitigation efforts. In 2021, the carbon price was set at 120€/t of CO_2_eq (**tCO_2_eq**) in Sweden and 45€/tCO_2_eq in France, whereas the EU European Trading System market price was 44€/tCO_2_eq (World Bank, 2021).

In this paper, we aim to provide insights into the design of PES schemes targeting the reduction of enteric methane emissions in dairy farms by examining 2 aspects of a payment mechanism for which failing to consider the feeding dimension could undermine its effectiveness in cutting enteric methane emissions: the choice of emissions indicator defining the environmental service, and the level of payment. By comparing an indicator constructed using a methodology applied in a real-life PES case study from France to the Tier 2 indicator currently used in the French annual GHG emissions inventory, which considers productivity only, we examine how the diet of dairy cows influences the enteric methane emissions attributed to farms. Changing the diet of cows to improve the milk fatty acid profile may generate additional production costs that are not yet evaluated. The second contribution is to quantify the additional cost of a change in the diet of cows at the farm level to evaluate the economic incentives needed for improving dairy systems toward more environment-friendly practices. We estimate the variable cost function of dairy farms at the scale of France and for different fodder systems.

### Enteric Methane Emission Indicators

Many indicators of enteric methane emissions have been developed and adapted to specific constraints, often related to the scale of their application and the data available for estimating emissions (Kebreab et al., 2006; Ellis et al., 2007; Negussie et al., 2017). For result-based PES, emissions should be regularly and easily monitored on farm. The emission indicator chosen must therefore be easily measurable by the farmers or the paying agent, or both, using a simple methodology that is also representative of the environmental target and reliable (based on strong and reliable scientific evidence; Allen et al., 2014). In addition, it must be sensitive to the different dimensions on which farmers can act to decrease emissions, in particular cows' diet and productivity.

The Intergovernmental Panel on Climate Change (IPCC) defines 3 families of methods, to be applied for national inventories according to data availability. Tier 1 methods attribute default yearly enteric methane emissions factors per dairy cow. Tier 1 methods provide aggregate estimates and are not adequate for monitoring changes over time and taking into account the variability of dairy farming practices. Tier 2 methods improve the accuracy of emission factors by including feed intake estimates of a representative diet and dairy cow (Gavrilova et al., 2019). Finally, Tier 3 methods require a precise characterization of cows' diet to account for digestibility. Both Tier 2 and Tier 3 approaches for France are presented in Eugène et al. (2019).

Several recent studies have applied these indicators. Stetter and Sauer (2022) applied a Tier 1 enteric methane emission indicator at the micro scale, but as part of an overall assessment of the relative GHG emissions and economic performance of farms. Life cycle assessments calculating the carbon footprint of dairy products tend to apply Tier 2 methods (Jayasundara et al., 2019; Wilkes et al., 2020), sometimes also including diet composition data (Hagemann et al., 2011; Gollnow et al., 2014). Bioeconomic models estimating emissions abatement costs in the agricultural sector use Tier 3 indicators of enteric methane emissions precise enough to capture both productivity and diet effects (Lengers et al., 2013; Mosnier et al., 2019). Most Tier 3 and “individualized” Tier 2 approaches mentioned above require a large amount of detailed individual farm-level data on cows' feed composition, making them too costly to be applied as monitoring indicators on a large number of farms. However, some Tier 3 methods can use information from milk analyses and, thus, can be more easily integrated into farm routines.

### A Tier-3 Indicator Applied in the French Eco-Methane Result-Based Scheme

Numerous studies have been carried out to understand the connection between dairy cows' enteric methane emissions, fat intake, and milk composition (Dong et al., 1997; Martin et al., 2006, 2008, 2010, 2011; Chilliard et al., 2009; Grainger and Beauchemin, 2011). In particular, experimental research shows that enteric methane emissions in grams of CH_4_ per liter (*Methane*) can be calculated from milk productivity in kilograms per cow per year (*Productivity*) and the ratio of the sum of fatty acids with 16 carbon atoms or fewer (*FA* ≤ *C*16) to the total amount of fatty acids (*Total FA*) in the milk they produce (Equation [1]). This ratio has a strong biological causal relationship with methanogenesis in the rumen and is significantly reduced by more sources of n-3 in cows' diet:

[1] $$Methane=11.368\times Productivity^{-0.4274}\times \frac{FA\leq C16}{TotalFA}\;.$$

This formula was coinvented by teams from the animal feed manufacturing company Valorex (P. Weill and G. Chesneau) and the French National Institute for Agricultural Research (INRA) (Y. Chilliard, M. Doreau and C. Martin), and received a patent under the title “Method for evaluating the quantity of methane produced by a dairy ruminant and method for decreasing and controlling such quantity” (WO2009156453A1; Weill et al., 2009). The necessary data to implement it are relatively easy to collect on farm. One needs milk productivity and an analysis of the fatty acid composition of milk, obtained via infrared spectroscopy. Milk infrared spectroscopy is relatively simple to integrate into the milk analysis routines of dairy farms and involves low costs. Because dairy farms already undertake milk analyses on a regular basis, it makes it a relatively cheap indicator to monitor emissions. In addition, the equation calculates enteric methane emissions per unit of product by taking into account both milk productivity and cows' feed, hence capturing both dimensions on which farmers can dedicate effort to reduce emissions.

The indicator described above has been used since 2011 to evaluate the reduction of enteric methane emissions of dairy farms participating in the PES scheme Eco-Methane in France, implemented and coordinated by the Bleu-Blanc-Coeur association (**BBC**). Eco-Methane meets the reference PES definition by Wunder (2015). In the scheme, private stakeholders (service users) give financial support to dairy farmers (service providers) who volunteer for actions that contribute to climate change mitigation (environmental services). The payment is conditional and proportional to the reduction of carbon dioxide equivalents (**CO_2_eq**), making the scheme a result-based PES. The reduction is calculated relative to baseline monthly emission levels attributed to the farm that are representative of its type of fodder system. Through the definition of different baseline scenarios, the PES design partially considers the variability in the potentiality of environmental services provision according to the production basin and the fodder system. Hence, rather than rewarding farms that produce the least emissions per unit of product (which would tend to favor the most productive farms), Eco-Methane supports all emission-reduction efforts.

The Eco-Methane scheme uses 11 scenarios of baseline emission levels representative of 11 fodder systems of French specialized dairy farms. These fodder systems were characterized by the French Dairy Interbranch Organization (CNIEL) in collaboration with the French Livestock Institute (IDELE) in 2009, based on large production basins (plains and mountainous areas), and the proportion of corn in fodder crop rotation systems (CNIEL, 2015). The baseline emissions for each scenario are determined by BBC using Eq. [1].

Eco-Methane brings together more than 600 farmers whose emissions reduction was estimated at 11% on average in 2017 (Bleu-Blanc-Coeur, 2022). The BBC pays farmers according to their reduction of methane emissions in CO_2_eq with a financial envelope made of donations from private actors (15€/tCO_2_eq on average in 2017). The main strengths of the scheme lie in the strong scientific foundations of the method for quantifying emissions and the easy participation procedure for dairy farmers. Each contract signatory commits to provide a monthly milk analysis to the association and to include feed with a high content of sources of n-3 in dairy cow rations (alfalfa, extruded linseed, grass). These data are used to estimate enteric methane emissions using Eq. [1]. Eco-Methane is recognized by the United Nations as a GHG emission-reduction project eligible for issuing carbon credits (UNFCCC, 2016).

## MATERIALS AND METHODS

Because no human or animal subjects were used, this analysis did not require approval by an Institutional Animal Care and Use Committee or Institutional Review Board.

### Data

Observations of a balanced panel of 735 French farms specialized in dairy milk production (OTEXE 45) from France's Farm Accountancy Data Network (**FADN**) for the years 2016 to 2018 were selected for the analysis. The FADN database is freely accessible online and is representative of socioeconomic and accountancy information of medium and large dairy farms, which contribute to more than 90% of the gross production and utilized agricultural area in France (Agreste, 2020). Due to this characteristic, it is a particularly relevant data set to investigate emission abatement costs at the national level. Because the compositions of the feed ration and milk are not surveyed, information on the diet of dairy cows is limited, and, in particular, the fatty acid profile is unknown. Instead, data on the fodder crop rotation systems are used to assess changes in crop rotation and approximate changes in feed composition. Descriptive statistics of the sample are presented in Table 1.

**Table 1:** Description of the sample (2,205 observations); source: the authors, based on 2016–2018 French FADN data (Agreste, 2020)

Variable	1st quartile	Median	Mean	3rd quartile
Utilized agricultural area^1^ (ha)	50.0	80.0	87.4	110.0
Fodder area^1^ (ha)	40.0	60.0	67.4	80.0
Corn silage area^1^ (ha)	1.0	10.0	14.1	20.0
Pasture area^1^ (permanent and temporary; ha)	26.0	40.0	50.3	61.0
Productivity (L/cow)	5,593.4	6,676.4	6,707.9	7,851.1
Number of dairy cows^1^	35	55	58	70
Agricultural work unit	1.0	2.0	1.8	2.1
Purchase of cattle feed concentrates (€)	14,326.0	24,996.5	32,853.2	43,645.0

1 Area variables and the number of dairy cows available in the database are ranges of values. We constructed the variables used in the analysis by taking the lower value of the range for each observation.

### Attribution of Enteric Methane Emissions

We use the Tier 2 method used in the French annual inventory of GHG emissions to define an indicator of enteric methane emissions sensitive to productivity effects. Dairy cows' emission factors are calculated from Eq. [2] (Citepa, 2020b):

[2] $$EF\;=0.0105\times \frac{Milk\;production}{Ncows}+48.971.$$

The emission factor *EF* (kg of CH_4_/cow per year) can be easily calculated for each farm of the FADN from the milk production (kg/yr) of the herd (*Milk production*) and the number of dairy cows (*Ncows*). The parameters of the formula are such that they are representative of breeding, feeding, and genetic conditions in France. Based on this calibration, the emission factor per cow varies according to milk productivity. We then derive an emission indicator (*TIER2*) per liter of milk (g of CH_4_/L), capturing variability according to milk productivity (L/cow per year):

[3] $$TIER2=\frac{EF\times 1,000}{productivity}.$$

Due to the absence of data on dairy cows' diet, a Tier 3 method cannot be applied to evaluate individual emissions of FADN farms and capture both productivity and diet effects. In particular, data are too limited to estimate individual enteric methane emissions of French farms using Eq. [1]. They are, however, sufficient to identify their Eco-Methane scenario and therefore the baseline emissions corresponding to their fodder system. Baseline emissions from the 11 scenarios are available per month and were obtained from BBC. We calculate the annual average to define the Eco-Methane baseline emissions indicator (Eco-Methane baseline, Table 2).

**Table 2:** Characteristics of the 11 baseline scenarios used in the Eco-Methane scheme; source: the authors, based on BBC data (Bleu-Blanc-Coeur, 2021)

Scenario	Corn in the fodder area	Production basin	Eco-Methane baseline (g of CH_4_/L)
1	More than 30%	Plains outside the western region	15.75
2		Plains of the western region	15.92
3	Between 10% and 30%	Plains outside the western region	15.83
4		Plains of the western region	16.43
5	Less than 10%	Plains outside the western region	16.56
6		Plains of the western region	17.38
7	More than 10%	Mountains	15.96
8	Less than 10%	Mountains of the Massif Central	17.13
9		Mountains of the Northern Alps	17.83
10		Mountains of Franche-Comté	16.22
11		Other mountains	17.20

An individual baseline scenario was assigned to each farm of the sample based on 2 criteria: the location and the share of corn silage in the fodder area of the farm. In the FADN database, the farm location variable corresponds to the 21 old French administrative regions (the administrative divisions were changed in 2015), whereas the Eco-Methane scenarios are defined according to large production basins built from a lower administrative level (departments). It was therefore necessary to allocate a production basin to each administrative region. For the regions with departments belonging to different production basins, we allocated the basin of the departments producing the highest volumes of milk to the entire region. This attribution was made using the 2018 annual dairy survey (Agreste, 2019) and is detailed in Appendix Table A1.

### Estimation of the Additional Cost of Milk Production with More Grass in the Fodder System

In France, the closest financial tool to a carbon tax is the Climate and Energy Contribution proportional to the carbon dioxide content of energy products (fossil fuels) (Rogissart et al., 2018). The contribution level was 30€/tCO_2_eq in 2017 and increased to 40€/tCO_2_eq in 2018 and 2019. Farmers participating in Eco-Methane received an average of 15€/tCO_2_eq in 2017, suggesting that the payment of the scheme is suboptimal and provides little incentive to participate (Bleu-Blanc-Coeur, 2022). Evaluating farmers' willingness to accept is necessary to define a more efficient price and trigger a large-scale adoption of practices decreasing enteric emissions.

Because the compositions of the feed rations and milk of cows are not available in the FADN, the effect of an improvement of the fatty acid profile on milk production costs cannot be analyzed directly. Instead, an evolution of the fodder crop rotation is assumed. As grass is a high source of n-3 fatty acids strongly encouraged in Eco-Methane, we assume that a commitment to the program would lead to an increase in grassland surfaces in farms. This hypothesis is quite strong and implies that the estimation of additional costs does not consider either the strategy of supplementing the ration with other feeds with high n-3 content such as extruded linseed or the optimization of grazing, increasing grass yield and quality without necessarily increasing grassland surfaces.

Based on dual-production theory (McFadden, 1978), we estimate a variable-cost function describing expenditures in variable production factors *x* with exogenous input prices *w* that minimize variable costs given the production level *y* targeted by the farmer and available quasi-fixed inputs *z*, such as land, labor, and equipment, that are assumed to be predetermined in the short term. The cost minimization approach is motivated by the fact that French dairy farmers are constrained in the quantity of milk they produce in the terms of their contract with dairy plants (Lambaré et al., 2018). This is confirmed in our data (see Appendix Table A2), as we observe a low variability of milk volumes across years for a same farm. The variable cost function (Eq. [4]) meets theoretical properties that we empirically check:

[4] $$VC\left(w,y,z\right)=\underset{x}{\min}wxsubject\;toy\leq f\left(x,z\right).$$

The variable cost function must be concave, nondecreasing, and homogeneous of degree 1 in variable input prices, decreasing with (binding) fixed factors of production, and monotonic according to output levels.

We estimate a system of equations comprising a homogeneous translog cost function (Eq. [5]) in which variable costs *VC* correspond to intermediate consumption and the variable input cost share functions (Eq. [6] and [7]), derived from Shephard's lemma. The translog functional form is commonly used in the literature on the cost structure and efficiency of dairy farms because of its flexibility and the possibility of imposing homogeneity of degree 1 (Moschini, 1988; Alvarez and Arias, 2003; Mosheim and Lovell, 2009; Nehring et al., 2009; Sobczyński et al., 2015; Tsionas et al., 2015; Singbo and Larue, 2016; Wimmer and Sauer, 2020). Variables *i* and *t* are indices for individuals and years, respectively. We assume that dairy farms produce one output, the quantity *Y_1_* of the milk of cows produced per year (milk production). Considering the joint production of milk and a bad output (enteric methane emissions) would be of main interest. This is the approach used in Njuki and Bravo-Ureta (2015) and Le et al. (2020), for instance. Implementing it in our study would require having enough information to compute an individualized enteric methane emission indicator capturing both cows' productivity and diets. The FADN data set does not provide enough information on cows' diets to compute the Eco-Methane indicator for each individual farm, and we cannot precisely compute the “bad” output.

We consider 2 variable inputs, fuel *X_1_* and cattle feedstuffs *X_2_*, for which the expenses represent a high share of intermediate consumption. The choice of including fuel is motivated by the possibility of calculating farm-level fuel prices and therefore capturing more heterogeneity. The price of fuel *W_1_* (fuel price) is calculated from the nonroad gas oil expenses and volumes. As individual cattle feedstuff prices are not available in the data, *W_2_* (feed price) is measured by the index of purchase prices of the means of agricultural production (IPAMPA) for adult cattle feeding stuffs of year *t* − 1, available for each French current administrative region. Although in practice, a bargaining effect may create endogeneity between milk production and feed prices, we assume it is sufficiently low not to overestimate too much milk marginal production costs (Duvaleix-Tréguer and Gaigné, 2016). Marginal costs tend to be overestimated when the adjustment of the feed unit prices to a change of farm size is not controlled for. In our case, capturing this endogeneity issue would require individual farm feed price data, information we do not have. Duvaleix-Tréguer and Gaigné (2016) found evidence of a price bargaining effect for hog production, but lower than the technology effect. For hog production, feed represents a larger share of the costs (about 60% of total cost) than for dairy production (25% of variable cost, see Appendix Table A2). Therefore, the price bargaining effect is expected to be lower for dairy farms.

We also include 3 quasi-fixed inputs. Grassland surface *Z_1_* (grassland) includes permanent and temporary pastures, alfalfa for dehydration, and other artificial fodders. We add 2 other quasi-fixed factors of production: machinery and constructions fixed assets *Z_2_* (capital) and annual work units *Z_3_* (labor). Because sample farms are all specialized in dairy milk production, we do not consider them as multi-output farms. We verify that the production of crops and other livestock products represents a much lower share of total gross products (see Appendix Table A2). However, we add the aggregated volume *Y_2_* of the other products of the farm (other productions) as a control variable to capture heterogeneity linked to diversification. *Y_2_* is calculated as the total gross product of the year (crop products, livestock products, and other products) net of animal purchases and milk production of the cows, deflated by the French agricultural producer price index of year *t*. We further control for the farm total utilized agricultural area *Q_1_* (utilized agricultural area), 20 regional dummies *D_ri_* approximating the pedoclimatic conditions, and time fixed effects with 2 yearly dummies *T_t_*. The coefficients *α*, *β*, *δ*, *ν*, *ρ*, *ζ*, *θ*, *γ*, and *µ* are the parameters to be estimated and describe the effects of the covariates on the independent variables; *e_it_*, *u_1it_*, and *u_2it_* are the error terms, assumed to follow a normal distribution:

[5] $$[\begin{array}{l} \ln\frac{VC_{it}}{W_{1it}}=\alpha_{0}+\beta_{1}\ln Y_{1it}+\alpha_{2}\ln\frac{W_{2it}}{W_{1it}}+\frac{1}{2}\alpha_{22}{\left(\ln\frac{W_{2it}}{W_{1it}}\right)}^{2}\\ +\sum_{h=1}^{3}\delta_{h}\ln Z_{hit}+\frac{1}{2}\beta_{11}\ln{Y_{1it}}^{2}\\ +\frac{1}{2}\sum_{h=1}^{3}\sum_{k=1}^{3}\delta_{hk}\ln Z_{hit}\ln Z_{kit}+\sum_{h=1}^{3}\nu_{2h}\ln\frac{W_{2it}}{W_{1it}}\ln Z_{hit}\\ +\sum_{h=1}^{3}\rho_{1h}\ln Y_{1it}\ln Z_{hit}+\zeta_{12}\ln Y_{1it}\ln\frac{W_{2it}}{W_{1it}}+\beta_{2}Y_{2it}+\theta_{1}Q_{1i}\\ +\sum_{r=1}^{20}\gamma_{r}D_{ri}+\sum_{t=16}^{17}\mu_{t}T_{t}+e_{t}; \end{array}$$

[6] $$\begin{array}{l} \frac{X_{1it}W_{1it}}{VC_{it}}=\\ 1-\alpha_{2}-\alpha_{22}\ln\frac{W_{2it}}{W_{1it}}-\overset{3}{\underset{h=1}{\sum }}\nu_{2h}\ln Z_{hit}-\zeta_{12}\ln Y_{1it}\;+u_{1it}; \end{array}$$

[7] $$\frac{X_{2it}W_{2it}}{VC_{it}}=\alpha_{2}+\alpha_{22}\ln\frac{W_{2it}}{W_{1it}}+\overset{3}{\underset{h=1}{\sum }}\nu_{2h}\ln Z_{hit}+\zeta_{12}\ln Y_{1it}+u_{2it}.$$

To account for the correlations between the error terms of the different equations, we use a 3-stage least squares regression analysis. The system of Eq. [5] + [6] + [7] is estimated for all the farms of the sample and then for the 3 major production basins and groups of Eco-Methane scenarios defined in Table 2 to identify potential differences in additional costs according to the type of dairy system. The different steps to derive the specification of the system are described in the Appendix. Descriptive statistics of the model variables of the sample and subsamples are presented in Table 3.

**Table 3:** Mean values of the model variables (SD in parentheses); source: the authors, based on 2016-2018 French FADN data (Agreste, 2020)

Variable^1^	France, n = 2,205	Western plains, n = 645	Plains outside the western region, n = 965	Mountains, n = 585	Plains, >30% of corn in the fodder area, n = 767	Plains, 10%–30% of corn in the fodder area, n = 574	Plains, <10% of corn in the fodder area, n = 279
Scenarios	1–11	2, 4, 6	1, 3, 5	7–11	1–2	3–4	5–6
Variable costs (€/yr)	128,073.5	139,405.3	136,399.4	96,533.0	170,654.1	124,656.0	90,248.5
	(105,612.7)	(107,167.1)	(112,800.6)	(80,772.0)	(124,937.8)	(71,877.3)	(57,036.7)
Milk production (L/yr)	398,594.1	446,128.0	402,355.2	306,703.0	526,707.0	396,281.9	156,204.9
	(297,513.2)	(328,781.5)	(296,923.0)	(226,416.1)	(333,580.8)	(213,270.9)	(266,216.2)
Other productions (€/base 100 per yr)	498.6	581.3	554.6	274.4	750.2	488.6	317.0
	(671.5)	(642.3)	(770.2)	(413.0)	(871.1)	(432.8)	(349.0)
Fuel price (€/L)	0.60	0.59	0.59	0.61	0.59	0.59	0.60
	(0.10)	(0.10)	(0.11)	(0.10)	(0.10)	(0.10)	(0.10)
Feed price (base 100)	96.6	96.5	96.6	96.8	96.6	96.5	96.6
	(2.4)	(2.4)	(2.4)	(2.3)	(2.4)	(2.4)	(2.4)
Grassland (ha)	51.1	42.1	52.7	65.5	34.2	50.6	66.1
	(41.0)	(31.5)	(38.6)	(47.8)	(25.8)	(32.5)	(36.1)
Capital (1,000€)	171.0	160.5	179.4	179.5	189.1	144.8	163.4
	(155.9)	(146.8)	(163.7)	(160.8)	(161.8)	(141.1)	(153.5)
Labor (AWU)	1.8	1.9	1.9	1.7	2.0	1.7	1.7
	(1.0)	(1.0)	(1.0)	(1.0)	(1.1)	(0.8)	(0.9)
UAA (ha)	87.4	84.3	92.6	86.3	93.3	85.5	79.3
	(58.1)	(55.3)	(60.0)	(57.8)	(60.6)	(48.2)	(42.1)

1 AWU = annual work unit; UAA = utilized agricultural area.

## RESULTS AND DISCUSSION

First, we present the allocation of the FADN dairy farms in the Eco-Methane scenarios, then we compare the 2 indicators Tier 2 and the Eco-Methane baseline. Finally, we discussed the additional costs associated with an increase in farm grassland area based on estimated marginal costs.

### Allocation of Eco-Methane Scenarios and Distinction of 3 Milk Production Basins

**Figure 1:**
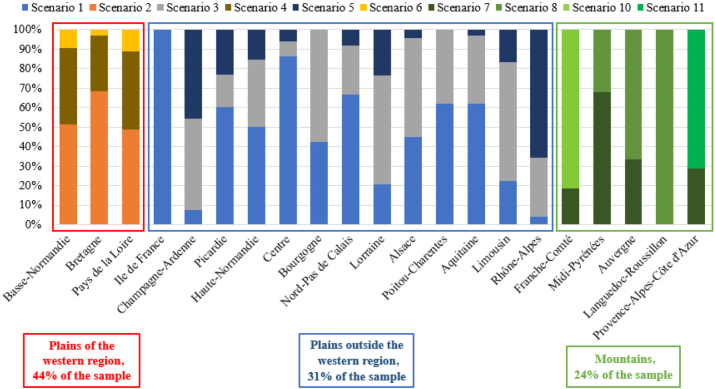
Distribution of Eco-Methane baseline scenarios among French old administrative regions. Source: the authors, based on 2016–2018 French FADN and BBC data (Agreste, 2020; Bleu-Blanc-Coeur, 2021).

Following the allocation of the Eco-Methane baseline scenarios to the farms of the sample, their proportion within each former administrative region can be observed (Figure 1). Farms in the regions of the western plains basin (Brittany, Pays de la Loire, and Basse-Normandie) represent 44% of the sample and are characterized by a strong dominance of corn silage and few grasslands in forage crop rotation systems. For example, 68% of farms in Brittany would be assigned scenario 2, with more than 30% of corn in the forage crop rotation, and 28% scenario 4, with 10% to 30%. The administrative regions of the plains production basin outside the western region (31% of the sample) and of the mountainous areas (24% of the sample) are quite different. Some plains regions, such as Rhône-Alpes, contain a high proportion of grazing systems dominated by grasslands (less than 10% of corn silage in the fodder crop rotation), whereas others, such as the Centre, have more intensive systems dominated by corn silage. All the observations from Languedoc-Roussillon correspond to grazing systems, whereas the observations from Midi-Pyrénées have a relatively small proportion (32%). Due to the missing information on departments in the FADN data set and our scenario allocation procedure, some farms have been allocated to a plain system scenario, when, in reality, they are located in a mountainous department and vice versa. This allocation might partly explain the large share of farms with grazing systems in Rhône-Alpes. Nevertheless, those farms produce relatively low volumes of milk in comparison with plain farms from the same region.

### Enteric Emissions: Relation to Productivity and Fodder System

The mean of the Tier 2 indicator for the sample is 18.5 g of CH_4_/L, whereas it is 16.3 g of CH_4_/L for the Eco-Methane baseline indicator, suggesting that considering the fodder cropping system in the calculation revises enteric emissions downward. Both indicators show a decrease in emissions per liter of milk as productivity increases (Table 4). Dairy systems with lower enteric emissions per liter of milk are productive systems with more than 30% of corn silage in the fodder crop rotation, whereas grass-based systems have the highest levels of emissions. The comparison of the 2 indicators allows us to nuance the performance of the different systems in terms of diet and productivity effects.

**Table 4:** Average enteric emissions of the sample according to the 2 indicators; source: the authors, based on 2016–2018 French FADN and BBC data (Agreste, 2020; Bleu-Blanc-Coeur, 2021)

Variable	Plains outside the western region				Western plains				Mountains		
Scenarios	1, 3, 5	1	3	5	2, 4, 6	2	4	6	7–11	7	8 to 11
% corn in fodder area		>30	30–10	<10		>30	30–10	<10		≥10	<10
Sample share (%)	31.4	10.0	9.9	11.5	44.3	23.7	16.8	3.8	24.3	6.5	17.8
Productivity (1,000 L/cow)	6.72	7.65	6.94	5.72	6.98	7.33	6.79	5.59	6.20	6.91	5.94
Tier 2 (g of CH_4_/L)	18.55^d^	17.46^l^	18.22fhj	19.78^a^	18.23ehi	17.80^k^	18.38defg	20.22^a^	19.07^c^	18.19gij	19.40^b^
Eco-Methane baseline (g of CH_4_/L)	16.07^q^	15.75^u^	15.83^t^	16.56^n^	16.24^p^	15.92^s^	16.43^o^	17.38^l^	16.55^n^	15.96^r^	16.76^m^

a–u Mean values with different superscripts differ (*P* < 0.05).

**Figure 2:**
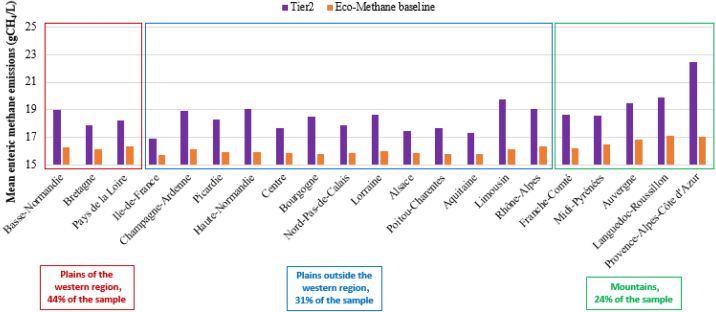
Average enteric emissions in French old administrative regions according to the 2 indicators. Source: the authors, based on 2016–2018 French FADN and BBC data (Agreste, 2020; Bleu-Blanc-Coeur, 2021).

**Figure A1:**
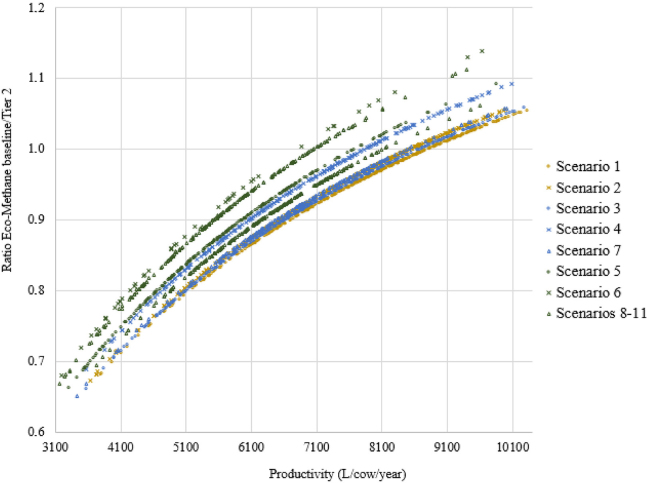
Ratio of the Eco-Methane baseline and Tier 2 enteric methane emissions indicators according to milk productivity for different Eco-Methane scenarios. Source: the authors, based on 2016–2018 French FADN and BBC data (Agreste, 2020; Bleu-Blanc-Coeur, 2021).

According to indicator Tier 2, farms in mountainous regions emit significantly more methane per liter of milk on average than farms in the plains, which can be explained by their lower productivity (*P* < 0.05). The same observation is true with the Eco-Methane indicator, but the difference between the groups is less (Table 4 and Figure 2). This finding shows that a diet effect partly compensates high emissions from low productivity in mountainous areas. The average share of grass in mountain fodder crop rotation systems is 90%, providing a diet rich in sources of n-3 to dairy cows and contributing to the mitigation of enteric emissions. In fact, we observe that the difference between the 2 indicators is large for low-productivity and grass-based dairy systems, and low for productive systems with a large share of corn silage in the fodder area (see Appendix Figure A1).

In plains areas, the average methane emissions per liter of milk of dairy farms with a high share of corn silage in the fodder area are higher in western regions (*P* < 0.05) because of lower productivity (Table 4). However, the difference between the 2 plains production basins is not significantly different for a fodder system with an intermediate share (scenario 3 vs. 4), or a low share (scenario 5 vs. 6) of corn silage. Therefore, computing enteric emissions with the Tier 2 indicator results in no significant difference between the 2 plains production basins for the same fodder system. However, the Eco-Methane baseline attributed to plains farms outside the western region is lower than in the western region for the same fodder system (*P* < 0.05). In western regions, farms have a significantly lower share of grass in the fodder rotation system (*P* < 0.01). This suggests that cows receive less n-3-rich fodder in their rations.

Our observations are in line with the literature (Martin et al., 2006; Grainger and Beauchemin, 2011). A recent meta-analysis of life cycle assessments also highlighted the negative relationships between milk yield and enteric methane emissions on the one hand and pasture intake and enteric methane emissions on the other hand (Lorenz et al., 2019). Other authors also show that enteric methane emissions particularly differ from one indicator to another in grazing systems (Hagemann et al., 2011). Choosing an adequate indicator of enteric methane emissions is the topic of ongoing debate. This analysis illustrates the importance of the choice of environmental indicator when designing a PES scheme targeting the reduction of enteric methane emissions in dairy farms, as it is likely to affect its environmental performance. We show the relevance of using an emission indicator sensitive to diet when defining economic incentives for the reduction of GHG emissions.

Detailed indicators are the most cost-effective, but due to heavy data collection needs (precise feed digestibility and composition), this advantage decreases when applied on a large scale (Lengers et al., 2013). An indicator such as the one used in Eco-Methane presents several advantages to be implemented on a large scale, and may be a better proxy compared with the most widely used Tier 2 and data-demanding Tier 3 indicators. It is precise enough to capture the efforts of farmers on both cow productivity and feed ration composition, and it considers the potential of an n-3-rich diet as a climate change mitigation practice. This feeding strategy is already implemented in dairy systems integrating a large share of grasslands in their fodder crop rotation systems, with the side provision of other environmental benefits (biodiversity maintenance). Additionally, the fatty acid composition of milk provides information on the complementary health benefits for consumers of an increase in sources of n-3 fatty acids in the diet of dairy cows (Weill et al., 2002). The accuracy of indicators based on milk analyses could be further improved by controlling for factors likely to affect the correlation between milk fatty acid composition and enteric emissions, such as the lactation stage (Negussie et al., 2017).

Furthermore, the environmental performance of PES schemes specifically targeting enteric methane emissions depends on the absence of negative spillovers on other factors of GHG emissions in dairy farms (e.g., fertilization management, machinery). On dairy farms, other important sources of GHG emissions are energy use, manure production and management, fertilizer production and application, and concentrate feed production (Chen and Holden, 2018; Jayasundara et al., 2019). Using more grass is a lever that can also contribute to lowering emissions from concentrate feed and to increasing carbon storage in agricultural soils (Mosnier et al., 2019; Wilkes et al., 2020). Moreover, dairy farms with more grasslands also tend to produce less GHG emissions in total at the farm level (Senga Kiessé et al., 2022). However, nitrous oxide emissions from soil can increase from more manure deposition on pasture (Del Prado et al., 2013; Wilkes et al., 2020). Decreasing enteric methane emissions per liter of milk is a lever particularly interesting to rapidly reduce the contribution of dairy farming to climate change while also considering the food security dimension as it refers to the quantity of food produced. However, it should be complementary to further farm-level and area-based assessments of GHG emissions to support effective mitigation strategies.

### Effects of Increasing Grassland Area on the Marginal Cost of Milk Production

Several model specifications were tested, and the results are robust to a change (single variable costs equation, system of equations with and without imposing constraints on the parameters to ensure homogeneity of degree 1). The estimation of variable input shares provides additional information and improves the quality of the variable cost estimation (measured by R^2^). Consistent with the hypothesis of cost minimization, imposing restrictions on the parameters across equations also improved the variable cost estimation quality. Therefore, we present the results of the constrained system estimation.

The variable cost functions estimated are homogeneous of degree 1 in variable input prices by specification, and we verified that they are concave and nondecreasing with input prices (positive estimated variable input cost shares), and monotonic in the milk production level. However, we observe that variable costs increase with the level of fixed factors of production for some observations (see Appendix Table A3). In particular, variable costs are decreasing with more capital for a significant share of farms in 4 models out of 7. Previous studies also found evidence of the violation of theoretical properties on quasi-fixed inputs, in particular capital (Mosheim and Lovell, 2009; Singbo and Larue, 2016; Wimmer and Sauer, 2020).

The first-order derivative of the variable cost function (Eq. [5]) gives the marginal cost function (Eq. [8]), in which parameter *ρ*_11_ corresponds to the effect of grassland surfaces on the marginal cost of milk:

[8] $$\frac{\partial VC_{it}}{\partial Y_{1it}}=\frac{VC_{it}}{Y_{1it}}\left(\beta_{1}+\beta_{11}\ln Y_{1it}+\overset{3}{\underset{h=1}{\sum }}\rho_{1h}\ln Z_{hit}+\zeta_{12}\ln\frac{W_{2it}}{W_{1it}}\right).$$

The results presented in the following paragraphs are calculated from the regression results detailed in Appendix Tables A4, A5, A6, A7, A8, A9, and A10.

When applied to all dairy farms of the sample and the subsamples of the western plains production basin and mountainous areas, the model suggests that producing milk with more grass does not significantly affect variable costs (Table 5). We find low but significant additional costs per additional hectare of grassland in plain dairy farms outside the western regions (+0.6€/1,000 L per ha; *P* < 0.10). This finding illustrates the natural competitive advantage that dairy farms from the western regions or mountainous areas have in producing more milk with grass, the former because of favorable temperate climate (Hennessy et al., 2020), the latter because of climatic and technical limitations for other land use (Coppa et al., 2019). A first implication for the design of a PES is that dairy farms from plains areas with a natural disadvantage for producing milk with grass require higher levels of economic incentives compared with other production basins. Other authors have also found evidence of differences in emissions abatement costs among dairy farms according to their geographical location (Njuki and Bravo-Ureta, 2015).

**Table 5:** Additional costs of milk production with an increase in grassland area; source: the authors, based on 2016–2018 French FADN and BBC data (Agreste, 2020; Bleu-Blanc-Coeur, 2021)

Variable	France	Plains of the western region	Plains outside the western region	Mountains	Plains, >30% of corn in the fodder area	Plains, 10%–30% of corn in the fodder area	Plains, <10% of corn in the fodder area
Marginal cost (€/1,000 L)	241.5	230.5	251.0	261.1	225.7	225.1	262.0
Additional cost (€/1,000 L/ha)	−0.02	−0.48	0.58†	−0.41	−1.38†	0.28	−1.82***
Variable cost regression R^2^	0.91	0.91	0.91	0.91	0.90	0.91	0.89

† *P* < 0.10,

*** *P* < 0.001.

Behind the heterogeneous additional costs found in plains production basins also lies a disparity depending on the type of fodder system (Table 5). Considering plains dairy farms, we compare those with a share of corn silage in the fodder area greater than 30%, between 10% and 30%, and less than 10%. Additional costs are significantly negative when the share of corn is higher than 30% (−1.4€/1,000 L per ha; *P* < 0.10) or lower than 10% (−1.8€/1,000 L per ha; *P* < 0.001), whereas they are not significant for an intermediate share between 10% and 30%. This second result reveals the economic opportunity of producing milk with more grass in terms of higher feed self-sufficiency (lower feed costs; Hanrahan et al., 2018). For plains grass-based systems with low milk productivity, achieving high self-sufficiency compensates otherwise relatively high marginal costs of production. For corn silage–based plains systems, reducing dependency on high-protein complements represents a notable source of cost savings. Current dominant intensive fodder systems involve high expenditures on specific corn inputs (e.g., seeds, herbicides) and high-protein complements (soy, rapeseed) to balance dairy cow feed rations. Synergies between the reduction of GHG emissions and the economic performance of intensive dairy farms have already been pointed out in the literature (Borreani et al., 2013; Jayasundara et al., 2019; Le et al., 2020). In contrast, no evidence of this economic opportunity is found for dairy systems with an intermediate share of corn silage in the fodder area. For those fodder systems, higher variable costs from an increase in energy consumption (machinery) and other expenses (seeds, fertilizers) related to additional pastures and alfalfa management may limit feed cost savings. A second implication of our findings is that, currently, a PES for producing milk with more grass with a low level of payment favors adoption by plains fodder systems with a large share of corn silage on the one hand, and fodder systems with a large share of grasslands on the other hand, over intermediate systems combining both corn silage and grass.

Our results suggest that the financial needs for dairy farms to incorporate more grass in their fodder crop rotation are different from one system to another. In particular, we identify the existence and magnitude of additional production costs linked to the modification of fodder crop rotations (increase in grassland area). In the prospect of designing PES such as the Eco-Methane program, and aiming to integrate the maximum number of existing farms into the scheme at the lowest cost, we provide evidence of the variability of dairy systems regarding their willingness to accept. With the perspective of accompanying dairy farms to trigger the adoption of mitigation practices at large scale, it is relevant to take into account this heterogeneity, for instance with a differentiated payment or a higher payment for all. Not considering the feeding strategy of dairy farmers, and in particular the type of fodder system, would lower the attractiveness of a payment scheme for some systems.

Given the low level of payment in current PES schemes (for instance in the Eco-Methane program), it seems reasonable to assume that participating farms already had good economic profitability or feeding practices, or both, compatible with emission reductions when entering the program. In the prospect of engaging more farmers and more liters of milk in the feed ration transition, we identify 3 types of dairy farms that could integrate a PES for the reduction of enteric methane emissions. Farms for which reducing enteric emissions is already profitable (negative additional costs, type 1), farms for which integrating such a PES system requires a financial incentive (no additional costs, type 2), and farms for which integrating the PES system requires high financial support (positive additional costs, type 3). Our study suggests that French plains-intensive farms broadly correspond to type 1. Although their individual willingness to accept such a PES scheme is likely to be low, the program would still need substantial financial means to offer a payment given the large number of units of emissions reductions to compensate (high milk productivity). Grass-based plains dairy systems also belong to type 1 farms, but their lower milk productivity involves lower units of emission reduction to compensate. In contrast, dairy farms located in mountainous areas are more likely to correspond to type 2, and require a payment level high enough to make the adoption of more grass-based diet the profitable option over keeping their current practices. For farms with an already high share of grasslands, increasing milk productivity may be an additional profitable lever to reduce enteric emissions per liter. We also classify plains dairy farms with 10% to 30% of corn silage in the fodder area as type 2 farms, involving a large number of emission-reduction units to compensate given the large number of potential participants and productivity levels. Finally, we identify plains dairy farms from outside the western region with a natural disadvantage in producing milk with more grass as belonging to type 3 on average. Although they represent the most expensive dairy farms to support, they are associated with lower enteric methane emissions per liter of milk according to the Eco-Methane baseline indicator. Higher payment levels would recognize the mitigation efforts they already provide.

However, in the long term, adapting payments to this heterogeneity might not lead to the most optimal allocation to minimize emissions per liter of milk and may introduce a distortion of competition in favor of the most polluting. An optimal scheme should equalize the marginal cost of methane abatement across all farms, for example through a tax on methane emissions. Knowledge of the heterogeneity of abatement costs can then be used to temporarily support the most polluting farms or with a natural disadvantage in improving their performance or in reorienting their activity if they are unable to compete with such a tax.

The positive effect of grass on methane emissions is likely to be partly offset by a reduction in the productivity of the cows. Other sources of n-3 fatty acids such as linseed could also be integrated in the feed ration to maintain productivity (Fuentes et al., 2008). However, feed complementation is likely to be a more expensive lever to implement for most farmers, as they are often not produced in farms. Due to absence of data, the additional costs of increasing the use of n-3-rich complements could not be estimated. Furthermore, for the Eco-Methane payment to efficiently subsidize the reduction of enteric methane emissions through an increase in grassland areas, the payment will have to cover both the additional costs per liter of milk and the other additional costs per hectare of grass. Beyond affecting production costs per unit of milk, a new hectare of grassland can have a direct effect on farm costs. In this study, we consider only variable costs (intermediate consumption). There may also be fixed costs (specific machinery for grass cultivation, buildings for storage) or other constraints (access to land), increasing the overall additional costs of participation. A study considering all farm costs (variable and fixed costs) found higher GHG emissions abatement costs per liter of milk in large farms (with a large number of dairy cows) compared with smaller farms (Njuki et al., 2016).

## CONCLUSIONS

Two aspects of PES design for reducing enteric emissions per liter of milk are particularly crucial to favor its environmental performance. First, the choice of emission indicator should measure farmers' efforts on both cows' productivity and diet, while being easily and regularly implemented on farm at low cost. Second, evaluating farmers' willingness to accept the program and its variability according to farm type is necessary to define the optimal payment level and the scheme's budget, ensuring sufficient participation. For a given productivity level, producing milk with more grass has a different effect on milk variable production costs depending on the production basin and fodder system. This research provides more insights into the influence of methane emissions reduction on the production costs of livestock farms, and how to improve support for pressing abatement measures and contribute effectively to achieving climate targets.

## Figures and Tables

**Table A1:** Eco-Methane scenarios attribution to the old French administrative regions using the 2018 annual dairy survey; source: the authors, based on the French 2018 annual dairy survey and BBC data (Agreste, 2019; Bleu-Blanc-Coeur, 2021)

Administrative region	Department	Scenarios	Production share (volume)	Attributed scenarios
Ile-de-France		1, 3, or 5		1, 3, or 5
Champagne-Ardennes		1, 3, or 5		1, 3, or 5
Picardie		1, 3, or 5		1, 3, or 5
Haute Normandie		1, 3, or 5		1, 3, or 5
Centre		1, 3, or 5		1, 3, or 5
Basse-Normandie		2, 4, 6		2, 4, or 6
Bourgogne		1, 3, or 5		1, 3, or 5
Nord-Pas-De-Calais		1, 3, or 5		1, 3, or 5
Lorraine	54	1, 3, or 5	59%	1, 3, or 5
	55	1, 3, or 5		
	57	1, 3, or 5		
	88	7 or 11	41%	
Alsace		1, 3, or 5		1, 3, or 5
Franche-Comté	25	7 or 10	76%	7 or 10
	39	7 or 10		
	70	1, 3, or 5	24%	
Pays de la Loire		2, 4, or 6		2, 4, or 6
Bretagne		1, 3, or 5		1, 3, or 5
Poitou-Charentes		1, 3, or 5		1, 3, or 5
Aquitaine		1, 3, or 5		1, 3, or 5
Midi-Pyrénées	46	7 or 8	60%	7 or 8
	12	7 or 8		
	9	1, 3, or 5	40%	
	31	1, 3, or 5		
	32	1, 3, or 5		
	65	1, 3, or 5		
	81	1, 3, or 5		
	82	1, 3, or 5		
Limousin		1, 3, or 5		1, 3, or 5
Rhône-Alpes	1	1, 3, or 5	75%	1, 3, or 5
	7	1, 3, or 5		
	26	1, 3, or 5		
	38	1, 3, or 5		
	42	1, 3, or 5		
	69	1, 3, or 5		
	73	7 or 8	25%	
	74			
Auvergne		7 or 8		7 or 8
Languedoc-Roussillon	11	1, 3, or 5	10%	7 or 8
	30	1, 3, or 5		
	34	1, 3, or 5		
	66	1, 3, or 5		
	48	7 or 8	90%	
Provence-Alpes-Côte d'Azur	5	7 or 11	87%	7 or 11
	4	1, 3, or 5	13%	
	6	1, 3, or 5		
	13	1, 3, or 5		
	83	1, 3, or 5		
	84	1, 3, or 5		

**Table A2:** Distribution of the variation of milk production volumes and the share of farm outputs in the total gross product; source: the authors, based on 2016-2018 French FADN (Agreste, 2020)^1^

Variable	Q1	Q2	Mean	Q3
% variation of milk production volume relative to the farm mean over 2016–2018	1.64	3.49	4.85	6.63
Share of milk production in the total gross product	69.19	77.74	76.57	85.15
Share of crop production in the total gross product	0.20	3.99	6.34	10.98
Share of other livestock products in the total gross product	0.00	0.00	0.87	0.00
Share of fuel expenses in intermediate consumption	3.60	4.60	4.88	6.00
Share of cattle feed expenses in intermediate consumption	19.10	25.20	24.63	31.00
Share of fertilizer expenses in intermediate consumption	3.29	5.29	5.78	7.76

1 Q1 = first quartile; Q2 = median; Q3 = third quartile.

**Table A3:** Violation of the theoretical properties of the variable cost function (%)

Theoretical property	France	Plains of the western region	Plains outside the western region	Mountains	Plains with more than 30% of corn silage in the fodder area	Plains with 10% to 30% of corn silage in the fodder area	Plains with less than 10% of corn silage in the fodder area
∂VC/∂VC∂Y1<0∂Y1<0	0.0	0.0	0.0	0.0	0.0	0.0	0.0
∂VC/∂VC∂Z1<0∂Z1<0	0.2	2.3	4.2	26.3	21.5	51.0	24.4
∂VC/∂VC∂Z2<0∂Z2<0	86.0	19.7	86.5	68.7	45.0	59.9	64.5
∂VC/∂VC∂Z3<0∂Z3<0	38.3	41.4	29.2	16.9	39.2	21.3	71.8
∂VC/∂VC∂W1>0∂W1>0	0.0	0.0	0.0	0.0	0.0	0.0	0.0
∂VC/∂VC∂W2>0∂W2>0	0.0	0.0	0.0	0.0	0.0	0.0	0.0
∂2VC/∂2VC∂W12<0∂W12<0	0.0	0.0	0.0	0.0	0.0	0.0	0.0
∂2VC/∂2VC∂W22<0∂W22<0	0.0	0.0	0.0	0.0	0.0	0.0	0.0

**Table A4:** Result of the system estimation for French dairy farms (n = 2,205; SE in parentheses); source: the authors, based on 2016–2018 French FADN (Agreste, 2020)

Coefficient	Estimate	Variable cost function	Fuel cost share function	Feed cost share function
$\alpha_{0}$	3.398*** (0.658)	Constant		
$\beta_{1}$	1.156*** (0.202)	Ln (milk production)		
$\alpha_{2}$	0.457*** (0.029)	Ln (feed price/fuel price)	1 – constant	Constant
$\delta_{1}$	0.007 (0.108)	Ln (grassland)		
$\delta_{2}$	0.086 (0.065)	Ln (capital)		
$\delta_{3}$	0.739*** (0.214)	Ln (labor)		
$\beta_{11}$	−0.054 (0.042)	0.5 × Ln (milk production)^2^		
$\rho_{11}$	−0.002 (0.020)	Ln (milk production) × Ln (grassland)		
$\rho_{12}$	−0.026† (0.015)	Ln (milk production) × Ln (capital)		
$\rho_{13}$	−0.151*** (0.040)	Ln (milk production) × Ln (labor)		
$\zeta_{12}$	0.024*** (0.003)	Ln (milk production) × Ln (feed price/fuel price)	−Ln (milk production)	Ln (milk production)
$\alpha_{22}$	0.002 (0.002)	0.5 × Ln (feed price/fuel price)^2^	−Ln (feed price/fuel price)	Ln (feed price/fuel price)
$\nu_{21}$	0.004* (0.002)	Ln (feed price/fuel price) × Ln (grassland)	−Ln (grassland)	Ln (grassland)
$\nu_{22}$	−0.003** (0.001)	Ln (feed price/fuel price) × Ln (capital)	−Ln (capital)	Ln (capital)
$\nu_{23}$	−0.009** (0.003)	Ln (feed price/fuel price) × Ln (labor)	−Ln (labor)	Ln (labor)
$\delta_{11}$	−0.014 (0.013)	0.5 × Ln (grassland)^2^		
$\delta_{12}$	0.001 (0.009)	Ln (grassland) × Ln (capital)		
$\delta_{13}$	−0.014 (0.023)	Ln (grassland) × Ln (labor)		
$\delta_{22}$	0.014* (0.007)	0.5 × Ln (capital)^2^		
$\delta_{23}$	0.035* (0.016)	Ln (capital) × Ln (labor)		
$\delta_{33}$	0.091 (0.061)	0.5 × Ln (labor)^2^		
$\beta_{2}$	0.000*** (0.000)	Other productions		
$\theta_{1}$	0.002*** (0.000)	Utilized agricultural area		
$\gamma_{1}$	−0.199 (0.326)	Champagne-Ardennes		
$\gamma_{2}$	−0.146 (0.325)	Picardie		
$\gamma_{3}$	−0.121 (0.325)	Haute Normandie		
$\gamma_{4}$	−0.086 (0.326)	Centre		
$\gamma_{5}$	−0.119 (0.325)	Basse-Normandie		
$\gamma_{6}$	−0.107 (0.329)	Bourgogne		
$\gamma_{7}$	−0.107 (0.325)	Nord-Pas-De-Calais		
$\gamma_{8}$	−0.119 (0.325)	Lorraine		
$\gamma_{9}$	−0.094 (0.327)	Alsace		
$\gamma_{10}$	−0.123 (0.325)	Franche-Comté		
$\gamma_{11}$	−0.156 (0.325)	Pays de la Loire		
$\gamma_{12}$	−0.207 (0.324)	Bretagne		
$\gamma_{13}$	−0.111 (0.326)	Poitou-Charentes		
$\gamma_{14}$	−0.080 (0.326)	Aquitaine		
$\gamma_{15}$	−0.257 (0.325)	Midi-Pyrénées		
$\gamma_{16}$	−0.200 (0.327)	Limousin		
$\gamma_{17}$	−0.104 (0.325)	Rhône-Alpes		
$\gamma_{18}$	−0.247 (0.325)	Auvergne		
$\gamma_{19}$	−0.233 (0.327)	Languedoc-Roussillon		
$\gamma_{20}$	−0.281 (0.332)	Provence-Alpes-Côte d'Azur		
		Baseline: Ile-de-France		
$\mu_{16}$	0.024* (0.011)	Year 2016		
$\mu_{17}$	0.003 (0.010)	Year 2017		
		Baseline: 2018		
R^2^		0.912	−306.535	−14.425

† *P* < 0.10

* *P* < 0.05,

** *P* < 0.01,

*** *P* < 0.001.

**Table A5:** Result of the system estimation for plain dairy farms in the western region (n = 645; SE in parentheses); source: the authors, based on 2016–2018 French FADN (Agreste, 2020)

Coefficient	Estimate	Variable cost function	Fuel cost share function	Feed cost share function
${\mathrm{alpha}}_{0}$	5.422*** (1.075)	Constant		
$\beta_{1}$	0.413 (0.419)	Ln (milk production)		
${\mathrm{alpha}}_{2}$	0.423*** (0.048)	Ln (feed price/fuel price)	1 – constant	Constant
$\delta_{1}$	0.423* (0.210)	Ln (grassland)		
$\delta_{2}$	−0.078 (0.146)	Ln (capital)		
$\delta_{3}$	0.744† (0.400)	Ln (labor)		
$\beta_{11}$	0.015 (0.100)	0.5 × Ln (milk production)^2^		
$\rho_{11}$	−0.046 (0.043)	Ln (milk production) × Ln (grassland)		
$\rho_{12}$	0.044 (0.038)	Ln (milk production) × Ln (capital)		
$\rho_{13}$	−0.100 (0.085)	Ln (milk production) × Ln (labor)		
$\zeta_{12}$	0.041*** (0.005)	Ln (milk production) × Ln (feed price/fuel price)	−Ln (milk production)	Ln (milk production)
$\alpha_{22}$	0.001 (0.004)	0.5 × Ln (feed price/fuel price)^2^	−Ln (feed price/fuel price)	Ln (feed price/fuel price)
$\nu_{21}$	−0.018*** (0.003)	Ln (feed price/fuel price) × Ln (grassland)	−Ln (grassland)	Ln (grassland)
$\nu_{22}$	−0.005* (0.002)	Ln (feed price/fuel price) × Ln (capital)	−Ln (capital)	Ln (capital)
$\nu_{23}$	0.003 (0.005)	Ln (feed price/fuel price) × Ln (labor)	−Ln (labor)	Ln (labor)
$\delta_{11}$	−0.047 (0.042)	0.5 × Ln (grassland)^2^		
$\delta_{12}$	0.004 (0.021)	Ln(grassland) × Ln(capital)		
$\delta_{13}$	0.030 (0.051)	Ln(grassland) × Ln(labor)		
$\delta_{22}$	−0.031† (0.018)	0.5 × Ln(capital)^2^		
$\delta_{23}$	−0.063* (0.032)	Ln(capital) × Ln(labor)		
$\delta_{33}$	0.143 (0.106)	0.5 × Ln(labor)^2^		
$\beta_{2}$	0.000*** (0.000)	Other productions		
$\theta_{1}$	0.002*** (0.000)	Utilized agricultural area		
$\gamma_{11}$	−0.043* (0.019)	Pays de la Loire		
$\gamma_{12}$	−0.099*** (0.019)	Bretagne		
		Baseline: Basse-Normandie		
$\mu_{16}$	0.003 (0.019)	Year 2016		
$\mu_{17}$	−0.018 (0.017)	Year 2017		
		Baseline: 2018		
R^2^		0.915	−362.617	−18.850

† *P* < 0.10

* *P* < 0.05, **P < 0.01,

*** *P* < 0.001.

**Table A6:** Result of the system estimation for plain dairy farms outside the western region (n = 975; SE in parentheses); source: the authors, based on 2016–2018 French FADN (Agreste, 2020)

Coefficient	Estimate	Variable cost function	Fuel cost share function	Feed cost share function
${\mathrm{alpha}}_{0}$	5.254*** (0.929)	Constant		
$\beta_{1}$	0.668* (0.293)	Ln (milk production)		
${\mathrm{alpha}}_{2}$	0.404*** (0.043)	Ln (feed price/fuel price)	1 – constant	Constant
$\delta_{1}$	−0.310* (0.156)	Ln (grassland)		
$\delta_{2}$	0.101 (0.087)	Ln (capital)		
$\delta_{3}$	1.558*** (0.321)	Ln (labor)		
$\beta_{11}$	0.012 (0.057)	0.5 × Ln (milk production)^2^		
$\rho_{11}$	0.047† (0.027)	Ln (milk production) × Ln (grassland)		
$\rho_{12}$	−0.038* (0.019)	Ln (milk production) × Ln (capital)		
$\rho_{13}$	−0.297*** (0.056)	Ln (milk production) × Ln (labor)		
$\zeta_{12}$	0.033*** (0.004)	Ln (milk production) × Ln (feed price/fuel price)	−Ln (milk production)	Ln (milk production)
$\alpha_{22}$	0.001 (0.004)	0.5 × Ln (feed price/fuel price)^2^	−Ln (feed price/fuel price)	Ln (feed price/fuel price)
$\nu_{21}$	0.009*** (0.002)	Ln (feed price/fuel price) × Ln (grassland)	−Ln (grassland)	Ln (grassland)
$\nu_{22}$	−0.001 (0.002)	Ln (feed price/fuel price) × Ln (capital)	−Ln (capital)	Ln (capital)
$\nu_{23}$	−0.032*** (0.005)	Ln (feed price/fuel price) × Ln (labor)	−Ln (labor)	Ln (labor)
$\delta_{11}$	−0.006 (0.016)	0.5 × Ln (grassland)^2^		
$\delta_{12}$	−0.001 (0.012)	Ln (grassland) × Ln (capital)		
$\delta_{13}$	−0.040 (0.031)	Ln (grassland) × Ln (labor)		
$\delta_{22}$	0.024* (0.009)	0.5 × Ln (capital)^2^		
$\delta_{23}$	0.087*** (0.022)	Ln (capital) × Ln (labor)		
$\delta_{33}$	0.022 (0.089)	0.5 × Ln (labor)^2^		
$\beta_{2}$	0.000*** (0.000)	Other productions		
$\theta_{1}$	0.001*** (0.000)	Utilized agricultural area		
$\gamma_{1}$	−0.175 (0.283)	Champagne-Ardennes		
$\gamma_{2}$	−0.100 (0.282)	Picardie		
$\gamma_{3}$	−0.078 (0.282)	Haute Normandie		
$\gamma_{4}$	−0.010 (0.283)	Centre		
$\gamma_{6}$	−0.067 (0.285)	Bourgogne		
$\gamma_{7}$	−0.079 (0.282)	Nord-Pas-De-Calais		
$\gamma_{8}$	−0.078 (0.282)	Lorraine		
$\gamma_{9}$	−0.059 (0.283)	Alsace		
$\gamma_{13}$	−0.052 (0.283)	Poitou-Charentes		
$\gamma_{14}$	−0.063 (0.283)	Aquitaine		
$\gamma_{16}$	−0.163 (0.285)	Limousin		
$\gamma_{17}$	−0.093 (0.282)	Rhône-Alpes		
		Baseline: Ile-de-France		
$\mu_{16}$	0.057** (0.017)	Year 2016		
$\mu_{17}$	0.017 (0.016)	Year 2017		
		Baseline: 2018		
R^2^		0.911	−276.665	−12.377

† *P* < 0.10

** *P* < 0.05,

** *P* < 0.01,

*** *P* < 0.001.

**Table A7:** Result of the system estimation for mountain dairy farms (n = 585; SE in parentheses); source: the authors, based on 2016–2018 French FADN (Agreste, 2020)

Coefficient	Estimate	Variable cost function	Fuel cost share function	Feed cost share function
${\mathrm{alpha}}_{0}$	1.397 (1.152)	Constant		
$\beta_{1}$	1.285** (0.427)	Ln (milk production)		
${\mathrm{alpha}}_{2}$	0.408*** (0.058)	Ln (feed price/fuel price)	1 – constant	Constant
$\delta_{1}$	514* (0.257)	Ln (grassland)		
$\delta_{2}$	0.270* (0.136)	Ln (capital)		
$\delta_{3}$	−0.193 (0.437)	Ln (labor)		
$\beta_{11}$	0.001 (0.094)	0.5 × Ln (milk production)^2^		
$\rho_{11}$	−0.060 (0.047)	Ln (milk production) × Ln (grassland)		
$\rho_{12}$	−0.055† (0.032)	Ln (milk production) × Ln (capital)		
$\rho_{13}$	−0.093 (0.083)	Ln (milk production) × Ln (labor)		
$\zeta_{12}$	0.025*** (0.006)	Ln (milk production) × Ln (feed price/fuel price)	−Ln (milk production)	Ln (milk production)
$\alpha_{22}$	0.006 (0.005)	0.5 × Ln (feed price/fuel price)^2^	−Ln (feed price/fuel price)	Ln (feed price/fuel price)
$\nu_{21}$	0.019*** (0.004)	Ln (feed price/fuel price) × Ln (grassland)	−Ln (grassland)	Ln (grassland)
$\nu_{22}$	−0.013*** (0.003)	Ln (feed price/fuel price) × Ln (capital)	−Ln (capital)	Ln (capital)
$\nu_{23}$	−0.016* (0.006)	Ln (feed price/fuel price) × Ln (labor)	−Ln (labor)	Ln (labor)
$\delta_{11}$	−0.062 (0.049)	0.5 × Ln (grassland)^2^		
$\delta_{12}$	−0.014 (0.020)	Ln (grassland) × Ln (capital)		
$\delta_{13}$	0.081 (0.055)	Ln (grassland) × Ln (labor)		
$\delta_{22}$	0.025* (0.012)	0.5 × Ln (capital)^2^		
$\delta_{23}$	0.078* (0.039)	Ln (capital) × Ln (labor)		
$\delta_{33}$	−0.074 (0.145)	0.5 × Ln (labor)^2^		
$\beta_{2}$	0.000*** (0.000)	Other productions		
$\theta_{1}$	0.002*** (0.000)	Utilized agricultural area		
$\gamma_{10}$	0.109*** (0.030)	Franche-Comté		
$\gamma_{18}$	0.006 (0.025)	Auvergne		
$\gamma_{19}$	0.032 (0.050)	Languedoc-Roussillon		
$\gamma_{20}$	0.017 (0.079)	Provence-Alpes-Côte d'Azur		
		Baseline: Midi-Pyrénées		
$\mu_{16}$	0.032 (0.023)	Year 2016		
$\mu_{17}$	0.030 (0.020)	Year 2017		
		Baseline: 2018		
R^2^		0.910	−289.950	−12.903

† *P* < 0.10

* *P < 0.05,

** *P* < 0.01,

*** *P* < 0.001.

**Table A8:** Result of the system estimation for plain dairy farms with more than 30% of corn in the fodder system (n = 767; SE in parentheses); source: the authors, based on 2016-2018 French FADN (Agreste, 2020)

Coefficient	Estimate	Variable cost function	Fuel cost share function	Feed cost share function
${\mathrm{alpha}}_{0}$	6.811*** (1.111)	Constant		
$\beta_{1}$	−0.265 (0.449)	Ln (milk production)		
${\mathrm{alpha}}_{2}$	0.388*** (0.046)	Ln (feed price/fuel price)	1 – constant	Constant
$\delta_{1}$	0.283 (0.233)	Ln (grassland)		
$\delta_{2}$	0.320* (0.140)	Ln (capital)		
$\delta_{3}$	1.445** (0.437)	Ln (labor)		
$\beta_{11}$	0.262* (0.105)	0.5 × Ln (milk production)^2^		
$\rho_{11}$	−0.092† (0.049)	Ln (milk production) × Ln (grassland)		
$\rho_{12}$	−0.078* (0.037)	Ln (milk production) × Ln (capital)		
$\rho_{13}$	−0.235* (0.096)	Ln (milk production) × Ln (labor)		
$\zeta_{12}$	0.046*** (0.006)	Ln (milk production) × Ln (feed price/fuel price)	−Ln (milk production)	Ln (milk production)
$\alpha_{22}$	0.002 (0.004)	0.5 × Ln(feed price/fuel price)^2^	−Ln (feed price/fuel price)	Ln (feed price/fuel price)
$\nu_{21}$	−0.013*** (0.003)	Ln (feed price/fuel price) × Ln (grassland)	−Ln (grassland)	Ln (grassland)
$\nu_{22}$	−0.007** (0.002)	Ln (feed price/fuel price) × Ln (capital)	−Ln (capital)	Ln (capital)
$\nu_{23}$	−0.008 (0.005)	Ln (feed price/fuel price) × Ln (labor)	−Ln (labor)	Ln (labor)
$\delta_{11}$	−0.001 (0.019)	0.5 × Ln (grassland)^2^		
$\delta_{12}$	0.063** (0.019)	Ln (grassland) × Ln (capital)		
$\delta_{13}$	−0.061 (0.040)	Ln(grassland) × Ln(labor)		
$\delta_{22}$	−0.005 (0.016)	0.5 × Ln(capital)^2^		
$\delta_{23}$	0.018 (0.031)	Ln(capital) × Ln(labor)		
$\delta_{33}$	0.237* (0.102)	0.5 × Ln(labor)^2^		
$\beta_{2}$	0.000*** (0.000)	Other productions		
$\theta_{1}$	0.002*** (0.000)	Utilized agricultural area		
$\gamma_{1}$	−0.194 (0.300)	Champagne-Ardenne		
$\gamma_{2}$	−0.241 (0.285)	Picardie		
$\gamma_{3}$	−0.236 (0.285)	Haute Normandie		
$\gamma_{4}$	−0.178 (0.286)	Centre		
$\gamma_{5}$	−0.214 (0.284)	Basse-Normandie		
$\gamma_{6}$	−0.163 (0.293)	Bourgogne		
$\gamma_{7}$	−0.185 (0.284)	Nord-Pas-De-Calais		
$\gamma_{8}$	−0.199 (0.287)	Lorraine		
$\gamma_{9}$	−0.217 (0.289)	Alsace		
$\gamma_{11}$	−0.228 (0.284)	Pays de la Loire		
$\gamma_{12}$	−0.273 (0.284)	Bretagne		
$\gamma_{13}$	−0.240 (0.287)	Poitou-Charentes		
$\gamma_{14}$	−0.178 (0.286)	Aquitaine		
$\gamma_{16}$	−0.236 (0.308)	Limousin		
$\gamma_{17}$	−0.120 (0.290)	Rhône-Alpes		
		Baseline: Ile-de-France		
$\mu_{16}$	0.034† (0.017)	Year 2016		
$\mu_{17}$	0.004 (0.016)	Year 2017		
		Baseline: 2018		
R^2^		0.902	−320.701	−17.652

† *P* < 0.10

* *P* < 0.05,

** *P* < 0.01,

*** *P* < 0.001.

**Table A9:** Result of the system estimation for plain dairy farms with 10% to 30% of corn in the fodder system (n = 574; SE in parentheses); source: the authors, based on 2016-2018 French FADN (Agreste, 2020)

Coefficient	Estimate	Variable cost function	Fuel cost share function	Feed cost share function
${\mathrm{alpha}}_{0}$	6.926*** (1.244)	Constant		
$\beta_{1}$	−0.008 (0.460)	Ln (milk production)		
${\mathrm{alpha}}_{2}$	0.426*** (0.051)	Ln (feed price/fuel price)	1 – constant	Constant
$\delta_{1}$	−0.071 (0.289)	Ln (grassland)		
$\delta_{2}$	0.100 (0.147)	Ln (capital)		
$\delta_{3}$	0.992* (0.445)	Ln (labor)		
$\beta_{11}$	0.083 (0.105)	0.5 × Ln (milk production)^2^		
$\rho_{11}$	0.031 (0.063)	Ln (milk production) × Ln (grassland)		
$\rho_{12}$	0.003 (0.035)	Ln (milk production) × Ln (capital)		
$\rho_{13}$	−0.190* (0.088)	Ln (milk production) × Ln (labor)		
$\zeta_{12}$	0.035*** (0.006)	Ln (milk production) × Ln (feed price/fuel price)	−Ln (milk production)	Ln (milk production)
$\alpha_{22}$	−0.001 (0.004)	0.5 × Ln (feed price/fuel price)^2^	−Ln (feed price/fuel price)	Ln (feed price/fuel price)
$\nu_{21}$	−0.004 (0.004)	Ln (feed price/fuel price) × Ln (grassland)	−Ln (grassland)	Ln (grassland)
$\nu_{22}$	−0.003 (0.002)	Ln (feed price/fuel price) × Ln (capital)	−Ln (capital)	Ln (capital)
$\nu_{23}$	0.009 (0.006)	Ln (feed price/fuel price) × Ln (labor)	−Ln (labor)	Ln (labor)
$\delta_{11}$	0.009 (0.060)	0.5 × Ln (grassland)^2^		
$\delta_{12}$	−0.023 (0.022)	Ln (grassland) × Ln (capital)		
$\delta_{13}$	−0.008 (0.063)	Ln (grassland) × Ln (labor)		
$\delta_{22}$	−0.002 (0.017)	0.5 × Ln (capital)^2^		
$\delta_{23}$	0.008 (0.035)	Ln (capital) × Ln (labor)		
$\delta_{33}$	0.076 (0.119)	0.5 × Ln (labor)^2^		
$\beta_{2}$	0.000*** (0.000)	Other productions		
$\theta_{1}$	0.001* (0.000)	Utilized agricultural area		
$\gamma_{1}$	−0.021 (0.053)	Champagne-Ardenne		
$\gamma_{2}$	0.024 (0.071)	Picardie		
$\gamma_{3}$	−0.039 (0.047)	Haute Normandie		
$\gamma_{4}$	0.130 (0.119)	Centre		
$\gamma_{5}$	−0.055† (0.029)	Basse-Normandie		
$\gamma_{6}$	−0.036 (0.066)	Bourgogne		
$\gamma_{7}$	−0.011 (0.045)	Nord-Pas-De-Calais		
$\gamma_{8}$	−0.020 (0.039)	Lorraine		
$\gamma_{9}$	0.004 (0.059)	Alsace		
$\gamma_{11}$	−0.063* (0.029)	Pays de la Loire		
$\gamma_{12}$	−0.163*** (0.029)	Bretagne		
$\gamma_{13}$	0.029 (0.053)	Poitou-Charentes		
$\gamma_{14}$	−0.038 (0.053)	Aquitaine		
$\gamma_{16}$	−0.037 (0.062)	Limousin		
		Baseline: Ile-de-France		
$\mu_{16}$	0.006 (0.019)	Year 2016		
$\mu_{17}$	−0.027 (0.018)	Year 2017		
		Baseline: 2018		
R^2^		0.908	−341.414	−18.898

† *P* < 0.10

* *P* < 0.05, **P < 0.01,

*** *P* < 0.001.

**Table A10:** Result of the system estimation for plain dairy farms with less than 10% of corn in the fodder system (n = 279; SE in parentheses); source: the authors, based on 2016-2018 French FADN (Agreste, 2020)

Coefficient	Estimate	Variable cost function	Fuel cost share function	Feed cost share function
${\mathrm{alpha}}_{0}$	−5.524* (2.228)	Constant		
$\beta_{1}$	2.652*** (0.786)	Ln (milk production)		
${\mathrm{alpha}}_{2}$	0.441*** (0.096)	Ln (feed price/fuel price)	1 – constant	Constant
$\delta_{1}$	2.645*** (0.552)	Ln (grassland)		
$\delta_{2}$	0.086 (0.210)	Ln (capital)		
$\delta_{3}$	−1.532* (0.731)	Ln (labor)		
$\beta_{11}$	−0.190 (0.172)	0.5 × Ln (milk production)^2^		
$\rho_{11}$	−0.269*** (0.080)	Ln (milk production) × Ln (grassland)		
$\rho_{12}$	−0.008 (0.054)	Ln (milk production) × Ln (capital)		
$\rho_{13}$	0.163 (0.157)	Ln (milk production) × Ln (labor)		
$\zeta_{12}$	0.039*** (0.010)	Ln (milk production) × Ln (feed price/fuel price)	−Ln (milk production)	Ln (milk production)
$\alpha_{22}$	−0.001 (0.009)	0.5 × Ln (feed price/fuel price)^2^	−Ln (feed price/fuel price)	Ln (feed price/fuel price)
$\nu_{21}$	−0.002 (0.008)	Ln (feed price/fuel price) × Ln (grassland)	−Ln (grassland)	Ln (grassland)
$\nu_{22}$	−0.002 (0.003)	Ln (feed price/fuel price) × Ln (capital)	−Ln (capital)	Ln (capital)
$\nu_{23}$	−0.039*** (0.011)	Ln (feed price/fuel price) × Ln (labor)	−Ln (labor)	Ln (labor)
$\delta_{11}$	−0.260* (0.118)	0.5 × Ln (grassland)^2^		
$\delta_{12}$	−0.055 (0.047)	Ln (grassland) × Ln (capital)		
$\delta_{13}$	0.280** (0.105)	Ln (grassland) × Ln (labor)		
$\delta_{22}$	0.037† (0.019)	0.5 × Ln (capital)^2^		
$\delta_{23}$	0.004 (0.057)	Ln (capital) × Ln (labor)		
$\delta_{33}$	−0.492*** (0.182)	0.5 × Ln (labor)^2^		
$\beta_{2}$	0.000*** (0.000)	Other productions		
$\theta_{1}$	0.002† (0.001)	Utilized agricultural area		
$\gamma_{1}$	−0.184** (0.060)	Champagne-Ardennes		
$\gamma_{2}$	−0.131† (0.076)	Picardie		
$\gamma_{3}$	−0.084 (0.086)	Haute Normandie		
$\gamma_{4}$	−0.108 (0.156)	Centre		
$\gamma_{5}$	0.028 (0.052)	Basse-Normandie		
$\gamma_{7}$	−0.249** (0.086)	Nord-Pas-De-Calais		
$\gamma_{8}$	−0.088 (0.054)	Lorraine		
$\gamma_{9}$	0.368† (0.208)	Alsace		
$\gamma_{11}$	−0.164*** (0.047)	Pays de la Loire		
$\gamma_{12}$	−0.060 (0.069)	Bretagne		
$\gamma_{14}$	−0.012 (0.191)	Aquitaine		
$\gamma_{16}$	−0.436** (0.158)	Limousin		
		Baseline: Ile-de-France		
$\mu_{16}$	0.006 (0.034)	Year 2016		
$\mu_{17}$	0.004 (0.030)	Year 2017		
		Baseline: 2018		
R^2^		0.894	−282.326	−9.027

† *P* < 0.10

* *P* < 0.05,

** *P* < 0.01,

*** *P* < 0.001.

## APPENDIX

Cost function with the translog specification:

$$\begin{array}{l} \ln VC\left(y,w,Z\right)=\alpha_{0}+\underset{r=1,\;2}{\sum }\beta_{r}\ln Y_{r}+\underset{i=1,2}{\sum }\alpha_{i}\ln w_{i}\\ +\underset{h=1,2,3}{\sum }\delta_{h}\ln Z_{h}+\beta_{11}\ln Y_{1}\ln Y_{1}\\ +\underset{i=1,2}{\sum }\underset{j=1,2}{\sum }\alpha_{ij}\ln w_{i}\ln w_{j}+\underset{h=1,2,3}{\sum }\underset{n=1,2,3}{\sum }\delta_{hn}\ln Z_{h}\ln Z_{n}\\ +\underset{i=1,2}{\sum }\zeta_{i1}\ln w_{i}\ln Y_{1}\\ +\underset{k=1,2,3}{\sum }\rho_{k1}\ln Z_{k}\ln Y_{1}+\underset{i=1,2}{\sum }\underset{k=1,2,3}{\sum }\nu_{ik}\ln w_{i}\ln Z_{k} \end{array}$$

Steps to impose homogeneity of degree 1 in input prices:

ln⁡VC(y,λw,X)=ln⁡λ+ln⁡VC(y,w,X)

$$\begin{array}{l} \ln VC\left(y,\lambda w,Z\right)\\ =\alpha_{0}+\underset{r=1,2}{\sum }\beta_{r}\ln Y_{r}+\underset{h=1,2,3}{\sum }\delta_{h}\ln Z_{h}\\ +\underset{i=1,2}{\sum }\alpha_{i}(\ln\lambda +\ln w_{i})+\beta_{11}\ln Y_{1}\ln Y_{1}\\ +\underset{i=1,2}{\sum }\underset{j=1,2}{\sum }\alpha_{ij}(\ln\lambda +\ln w_{i})(\ln\lambda +\ln w_{j})\\ +\underset{h=1,2,3}{\sum }\underset{n=1,2,3}{\sum }\delta_{hn}\ln Z_{h}\ln Z_{n}\\ +\underset{i=1,2}{\sum }\zeta_{i1}\left(\ln\lambda +\ln w_{i}\right)\ln Y_{1}+\underset{k=1,2,3}{\sum }\rho_{k1}\ln Z_{k}\ln Y_{1}\\ +\underset{i=1,2}{\sum }\underset{k=1,2,3}{\sum }\nu_{ik}\left(\ln\lambda +\ln w_{i}\right)\ln Z_{k} \end{array}$$

$$\begin{array}{l} \ln VC\left(y,\lambda w,Z\right)\\ =\ln VC\left(y,w,Z\right)+\underset{i=1,2}{\sum }\alpha_{i}\ln\lambda +\underset{i=1,2}{\sum }\underset{j=1,2}{\sum }\alpha_{ij}{(\ln\lambda }^{)2}\\ +\underset{i=1,2}{\sum }\underset{j=1,2}{\sum }\alpha_{ij}\ln\lambda \ln w_{j}+\underset{i=1,2}{\sum }\underset{j=1,2}{\sum }\alpha_{ij}\ln\lambda \ln w_{i}\\ +\underset{i=1,2}{\sum }\zeta_{i1}\ln\lambda \ln Y_{1}+\underset{i=1,2}{\sum }\underset{k=1,2,3}{\sum }\nu_{ik}\ln\lambda \ln Z_{k} \end{array}$$

Parameter constraints to impose on our system:

$$\begin{array}{l} \underset{i=1,2}{\sum }\alpha_{i}=1\;;\;\forall i\;\in \left(1,2\right),\underset{j=1,2}{\sum }\alpha_{ij}=0;\forall \left(i,j\right)\in \left(1,2\right),\\ \alpha_{ij}=\alpha_{ji}\;;\;\underset{i=1,2}{\sum }\zeta_{i1}=0\;;\;\forall k\;\in \left(1,2,3\right),\;\underset{i=1,2}{\sum }\nu_{ik}=0 \end{array}$$

Homogeneous function to estimate:

$$\begin{array}{l} \ln\frac{VC\left(y,w,Z\right)}{w_{1}}=\alpha_{0}+\underset{r=1,\;2}{\sum }\beta_{r}\ln Y_{r}+\alpha_{2}\ln\frac{w_{2}}{w_{1}}+\underset{h=1,2,3}{\sum }\delta_{h}\ln Z_{h}\\ +\beta_{11}\ln Y_{1}\ln Y_{1}\\ +\alpha_{22}(\ln{\frac{w_{2}}{w_{1}}}^{)2}+\underset{h=1,2,3}{\sum }\underset{n=1,2,3}{\sum }\delta_{hn}\ln Z_{h}\ln Z_{n}+\zeta_{12}\ln Y_{1}\left(\ln\frac{w_{2}}{w_{1}}\right)\\ +\underset{k=1,2,3}{\sum }\rho_{k1}\ln Z_{k}\ln Y_{1}+\underset{k=1,2,3}{\sum }\nu_{2k}\ln\frac{w_{2}}{w_{1}}\ln Z_{k} \end{array}$$

Input demand functions, applying Shephard's lemma:

$$\frac{\partial VC\left(y,w,Z\right)}{\partial w_{i}}=X_{i}$$

$$\begin{array}{l} \frac{\partial \ln VC\left(y,w,Z\right)}{\partial \ln w_{i}}=\frac{\partial VC\left(y,w,Z\right)}{\partial w_{i}}\frac{w_{i}}{VC\left(y,w,Z\right)}=\\ \frac{X_{i}w_{i}}{VC\left(y,w,Z\right)}=S_{i} \end{array}$$

$$S_{1}=1-\alpha_{2}-2\alpha_{22}\ln\frac{w_{2}}{w_{1}}-\zeta_{12}\ln Y_{1}-\underset{k=1,2,3}{\sum }\nu_{2k}\ln Z_{k}$$

$$S_{2}=\alpha_{2}+2\alpha_{22}\ln\frac{w_{2}}{w_{1}}+\zeta_{12}\ln Y_{1}+\underset{k=1,2,3}{\sum }\nu_{2k}\ln Z_{k}$$
